# ELMO-CPAP: an effective approach in the management of patients with acute hypoxemic respiratory failure

**DOI:** 10.36416/1806-3756/e20240349

**Published:** 2024-11-16

**Authors:** Erich Vidal Carvalho, Lídia Maria Carneiro da Fonseca, Bruno Valle Pinheiro

**Affiliations:** 1. Unidade de Terapia Intensiva, Hospital Universitário, Universidade Federal de Juiz de Fora, Juiz de Fora (MG) Brasil.; 2. Departamento de Clínica Médica, Faculdade de Medicina, Universidade Federal de Juiz de Fora, Juiz de Fora (MG) Brasil.; 3. Centro de Biologia da Reprodução, Universidade Federal de Juiz de Fora, Juiz de Fora (MG) Brasil.

The benefits of non-invasive ventilation (NIV) are well established in the treatment of exacerbations of COPD and cardiogenic acute pulmonary edema, conditions in which the application of NIV can reduce rates of endotracheal intubation and mortality.^1^ However, in cases of acute hypoxemic respiratory failure (AHRF), particularly in ARDS, the use of NIV remains a subject of controversy. The European Respiratory Society/American Thoracic Society (ERS/ATS) guidelines for NIV in acute respiratory failure, for instance, do not provide a recommendation regarding the use of NIV in AHRF, citing the uncertainty of the evidence.^2^ A similar position is found in the guidelines from the European Society of Intensive Care Medicine on ARDS, although, in that document, the authors suggest the use of CPAP over conventional oxygen therapy in patients with AHRF due to COVID-19.^3^

Several concerns have been raised regarding the use of NIV in AHRF. The primary concern relates to delays in the decision to proceed with endotracheal intubation, which is associated with an increased risk of mortality.^4^ Another concern involves the administration of high tidal volumes, particularly when two levels of positive pressure are employed, as this may increase the inflammatory lung injury and worsen prognosis.^5^ Additionally, many patients do not tolerate, for prolonged periods, higher levels of positive pressure, which are theoretically desired in cases of moderate to severe hypoxemia, thereby limiting the benefits of NIV.^2^ ^,^ ^3^

The application of NIV via a helmet interface may overcome some of these limitations. The helmet interface enhances patient tolerance to elevated PEEP levels and allows prolonged application of NIV, which improves oxygenation. Furthermore, higher PEEP levels increase functional residual capacity and reduce ventilatory inhomogeneity, which might prevent the occurrence of ventilator-induced lung injury (VILI).^6^ In a randomized clinical trial, Grieco et al. demonstrated that patients with moderate to severe AHRF due to COVID-19 who received NIV through a helmet interface presented lower rates of tracheal intubation, compared with those who received high-flow nasal oxygen.^7^ In a previous randomized clinical trial, Patel et al. compared NIV delivered by a helmet with NIV delivered by face mask in patients with non-COVID-19 ARDS. They found that patients who received NIV through the helmet interface presented lower intubation rates (18.2% vs. 61.5% in the face mask group), and lower mortality at 90 days (34.1% vs. 56.4% in the face mask group).^8^

The results of these two studies are in line with those of a network meta-analysis^9^ that evaluated randomized clinical trials including patients with AHRF and compared the following treatments: high-flow nasal oxygen, face mask NIV, helmet NIV, or standard oxygen therapy. That meta-analysis found that helmet NIV was associated with a lower risk of endotracheal intubation when compared with standard oxygen therapy, high-flow nasal oxygen, and face mask NIV. Additionally, helmet NIV was also associated with a lower risk of mortality compared with the other three treatments.^9^

In this issue of the *Jornal Brasileiro de Pneumologia*, Matos et al. present their experience with the application of CPAP in patients with AHRF due to COVID-19 through a new helmet interface, known as ELMO, in a retrospective cohort study.^10^ The ELMO interface was developed in the of state of Ceará, Brazil, during the COVID-19 pandemic and allows the application of CPAP levels ranging from 6 to 15 cmH_2_O, with a FIo_2_ of up to 1. Positive pressure is generated by two compressed air flow meters and a PEEP valve adjusted to an air outlet, allowing the application of CPAP without the need for a ventilator. The lack of necessity for a ventilator constituted a significant advantage during the pandemic, when the number of mechanical ventilation devices available was insufficient. Furthermore, the ELMO-CPAP system facilitates the application of NIV outside the ICU.

The results obtained by Matos et al.^10^ were consistent with the existing literature. The endotracheal intubation rate among the 180 patients who received ELMO-CPAP was 27.2%, and the in-hospital mortality rate was 18.9%. Among the nonintubated patients (n = 131), the mortality rate was 3.1%, while it was 61.2% among the intubated patients (n = 49). The high mortality rate among patients who are intubated following failure of NIV continues to be a challenge in the management of AHRF and highlights the need to avoid delays in endotracheal intubation. In this context, recognizing patients at a higher risk of failure is essential. The authors identified several factors associated with a higher risk of failure, including those related to greater severity (advanced age and pulmonary involvement on chest CT greater than 75%) and those related to the response to ELMO-CPAP application (ROX index after two hours of NIV and duration of the first NIV session < 24 h). These findings highlight the importance of closely monitoring patients undergoing NIV and providing appropriate ventilatory support for extended durations, conditions that require a trained and experienced team.

Another important finding in the study by Matos et al.^10^ was the good tolerance of patients to ELMO-CPAP, which allowed for the application of NIV for prolonged periods, with a median duration of 39 h during the first session (IQR = 24-48 h). This tolerance to ELMO-CPAP, also described in other studies employing a helmet interface for NIV application, may have been facilitated by the judicious use of sedation by the team. The management of sedation also requires training and expertise to ensure that it does not compromise clinical assessment and early identification of failure in noninvasive ventilatory support.

In conclusion, the findings of this retrospective cohort study, consistent with recent literature, confirm that the appropriate application of NIV with a helmet interface is an effective approach in the management of patients with AHRF.
